# Efficacy of acidified water-in-oil emulsions against desiccated *Salmonella* as a function of acid carbon chain-length and membrane viscosity

**DOI:** 10.3389/fmicb.2023.1197473

**Published:** 2023-06-12

**Authors:** Shihyu Chuang, Mrinalini Ghoshal, Lynne McLandsborough

**Affiliations:** ^1^Department of Food Science, University of Massachusetts, Amherst, MA, United States; ^2^Department of Microbiology, University of Massachusetts, Amherst, MA, United States

**Keywords:** cleaning and sanitation, desiccated *Salmonella*, dry food processing, membrane viscosity, oil-based antimicrobial delivery

## Abstract

Sanitizing low-moisture food (LMF) processing equipment is challenging due to the increased heat resistance of *Salmonella* spp. in low-water activity (a_w_) environments. Food-grade oils mixed with acetic acid have been shown effective against desiccated *Salmonella*. In this study, different hydrocarbon chain-length (C_n_) organic acids were tested against desiccated *Salmonella* by using 1% v/v water-in-oil (W/O) emulsion as the delivery system for 200 mM acid. Fluorescence lifetime imaging microscopy (FLIM) was utilized with a BODIPY-based molecular rotor to evaluate membrane viscosity under environmental conditions such as desiccation and temperature elevation. Drying hydrated *Salmonella* cells to 75% equilibrium relative humidity (ERH) increased the membrane viscosity from 1,199 to 1,309 mPa·s (cP) at 22°C. Heating to 45°C decreased the membrane viscosity of hydrated cells from 1,199 to 1,082 mPa·s, and decreased that of the desiccated cells from 1,309 to 1,245 mPa·s. At both 22°C and 45°C, desiccated *Salmonella* was highly susceptible (>6.5 microbial log reduction (MLR) per stainless-steel coupon) to a 30-min treatment with the W/O emulsions formulated with short carbon chain acids (C_1-3_). By comparison, the emulsion formulations with longer carbon chain acids (C_4-12_) showed little to no MLR at 22°C, but achieved >6.5 MLR at 45°C. Based upon the decreased *Salmonella* membrane viscosity and the increased antimicrobial efficacy of C_4-12_ W/O emulsions with increasing temperature, we propose that heating can make the membrane more fluid which may allow the longer carbon chain acids (C_4-12_) to permeate or disrupt membrane structures.

## 1. Introduction

Due to the survival kinetics of *Salmonella* under desiccation, this organism is of particular concern across the manufacturing systems of low-moisture foods (LMFs) such as chocolate, peanut butter, and dry pet foods (Gruzdev et al., 2011; Beuchat et al., 2013; Kępińska-Pacelik and Biel, 2021). Recently, *Salmonella* contamination associated with chocolates has prompted a series of recall and regulatory inspection causing a plant shutdown for months and an economic loss of millions to the manufacturer (Whitworth, 2022). Traditional wet cleaning approaches are not ideal for LMF processing due to the immiscible nature of water and lipids, and that the water residing on the processing equipment can induce microbial growth (Moerman and Mager, 2016). Commercial peanut butter and chocolate processing lines are usually cleaned by flushing with heated oil. Alcohol-based agents are used for sanitization as these compounds can evaporate completely leaving no residue on the contact surface (Grasso et al., 2015). However, since alcohol is flammable, processing shutdown is required for equipment cooldown before sanitization. For this reason, a dry sanitation system that can be applied with temperature elevation would be advantageous.

Oils mixed with food-grade organic acids have been shown to be an effective means for tackling desiccated *Salmonella* (Ghoshal et al., 2022; Chuang et al., 2023). In these reports, synergistic microbial inactivation was achieved by applying acetic acid-acidified oil combined with heating and water-in-oil (W/O) emulsion. Structure-wise, organic acids are a carboxylic group (polar) attached to an aliphatic chain of varying hydrocarbon chain-lengths (nonpolar), rendering these compounds preferentially soluble within a binary system consisting of an aqueous phase and an organic phase. For instance, since acetic acid has a partition coefficient (log *P*) of −0.17 at 25°C, its location within a W/O emulsion colloid is a dynamic motion where the acid mostly remains in the water phase yet being capable of partitioning into the oil phase (Chuang et al., 2023). Depending on the acid carbon chain-length, this motion can change to where more hydrophobic acids (e.g., with log *P* > 0) would mostly remain in oil yet being able to partition into water. The role of W/O emulsion in accelerating microbial inactivation from acidified oil has been linked to several physiological and physicochemical phenomenon such as membrane disruption (Ghoshal et al., 2022), the diffusion and partitioning of acid due to preferential affinity (Chapman and Ross, 2009; Trček et al., 2015; Bertheussen et al., 2017), and osmotic lysis in the presence of unbalanced vapor pressure at the oil-cell interface (Syamaladevi et al., 2016; Xie et al., 2021; Chuang et al., 2023). The dispersion of water in oil allows the partitioning of acetic acid from the continuous oil phase to the water droplets which subsequently functions as the vehicle for the acid to enter the cytoplasm (Chuang et al., 2023).

Researchers have studied the gene expression of *Salmonella* under low-a_w_ conditions, reporting several biological processes that were involved in the stress response, such as osmoregulation, heat-shock protein synthesis, and fatty-acid metabolism (Gruzdev et al., 2012; Chen et al., 2013; Fong and Wang, 2016; Chen and Jiang, 2017). However, more evidence in the fundamental physiology of desiccated *Salmonella* is needed to support the development of an effective sanitation method. Bacterial cells can adjust membrane viscosity upon environmental fluctuations allowing adaptation (Stein, 2012; Ernst et al., 2016). Viscosity is defined as the resistance of a fluid to deformation at a given rate, which can be mechanically assessed by shearing with liquids assuming homogeneity within a system large in volume. However, measurements will be prone to variation if mechanically assessed at the scale of a micron, such as bacterial plasma membranes, where systemic homogeneity cannot be assumed. Due to the hydrophobic nature, 4,4-difluoro-5,7-dimethyl-4-Bora-3a,4a-diaza-s-indacene (BODIPY)-based molecular rotors localize in certain biological regions such as the lipid bilayer (Kuimova, 2012). Molecular rotors are synthetic fluorophores that, following energy absorption from a certain wavelength of light, can emit photons *via* fluorescent or nonfluorescent pathways. Nonradioactive decay of the activated fluorophore occurs through intramolecular rotation which is dependent upon environmental viscosity. Thus, the fluorescence signal of a molecular rotor increases with increasing viscosity of where it localizes. Fluorescence lifetime imaging microscopy (FLIM) measures the time it takes an excited fluorophore to return to the ground state (Datta et al., 2020). The lifetime of a fluorophore is independent from the local fluorophore concentration and thus can be used for measuring micro-viscosity (Castellani et al., 2020). Application of BODIPY with FLIM allows an effective means to acquire numerical indications of the viscosity of bacterial plasma membrane.

The objective of this study was to evaluate the membrane viscosity of desiccated *Salmonella* and its sensitivity to treatment with W/O emulsions loaded with different chain-length organic acids, with temperature elevation.

## 2. Materials and methods

### 2.1. Bacterial strains and inoculum preparation

*Salmonella enterica* subsp. *enterica* serovars were obtained from the American Type Culture Collection (ATCC, Manassas, VA). These included *S.* Enteritidis phage type 30 (BAA-1045, an outbreak strain associated with LMF processing), and three other strains recommended for testing produce sanitizers (Harris et al., 2001), i.e., *S*. Michigan (BAA-709), *S*. Montevideo (BAA-710), and *S*. Gaminara (BAA-711). Stock cultures were stored at −80°C in tryptic soy broth (TSB, Difco, Becton Dickinson, Sparks, MD) with 25% glycerol (#G7893, Sigma-Aldrich, St. Louis, MO). Working cultures were prepared by streaking stock cultures on tryptic soy agar (TSA, Difco, Becton Dickinson) with overnight incubation at 37°C, which were maintained at 4°C and replaced monthly. Inoculum was prepared following the procedure by Uesugi et al. (2006). Prior to each experiment, an isolated colony was transferred from the working culture to TSB (20 mL) and incubated at 37°C for 24 h. To produce bacterial lawns, 100 μL of this liquid culture was spread onto TSA supplemented with 0.6% yeast extract (TSAYE, #BP1422, Fisher Scientific, Pittsburgh, PA) with incubation at 37°C for 24 h. The sessile cells were harvested using sterile scrapers (Fisher Scientific) and resuspended in distilled water (18.2 MΩ·cm, Direct-Q Water Purification System, Merck KgaA, Darmstadt, Germany). The optical density at 600 nm (OD_600_) was adjusted to 1.2 to achieve approximately 10^9^ CFU/mL as the inoculum. For cocktail studies, this procedure was repeated with each strain and the inocula were combined as a cocktail.

### 2.2. Desiccation of bacteria

Aliquots (20 μL) of the inoculum were added to stainless-steel coupons (2B-finish, 12.7 mm diameter × 3.8 mm thickness, Biosurface, Bozeman, MT) and held in a desiccator at room temperature (22°C) for 20 h. A saturated solution of sodium chloride (#S271-1, Fisher Scientific) was used to maintain the equilibrium relative humidity (ERH) at 75%. This was based upon our previous report that the cells desiccated at 75% ERH were more resistant to treatment with acidified W/O emulsions than those desiccated at 33% ERH (Chuang et al., 2023). The time of desiccation was based on a report by Gruzdev et al. that cellular dehydration reached the maximum level after 20 h of desiccation (Gruzdev et al., 2011). Prior to each experiment, the coupons were washed with soapy water, soaked in acetone overnight, rinsed with distilled water, autoclaved, and dried for use.

### 2.3. Preparation of acidified W/O emulsion

#### 2.3.1. Formulation and emulsification method

Commercially available vegetable oils (canola oil, corn oil, and peanut oil) were used interchangeably as the carrier oil within the tested systems. The types of carrier oil did not influence the antimicrobial efficacy of acidified oils and W/O emulsions (Ghoshal et al., 2022; Chuang et al., 2023). Polyglycerol polyricinoleate (PGPR 4150, Palsgaard, Juelsminde, Denmark), a food-grade hydrophobic surfactant, was dissolved in the carrier oil at 3% w/w. This oil-surfactant stock solution was mixed with organic acids (≥ 97%, Sigma-Aldrich) using a nutating mixer (Orbitron Rotator II Model 260,250, Boekel Scientific, Feasterville-Trevose, PA) at room temperature for 1 h to make acidified oils at 200 mM as calculated based on the sum of the volumes of the oil, surfactant, and acid. The organic acids were of different hydrocarbon chain-lengths (C_n_) including formic/methanoic acid (C_1_), acetic/ethanoic acid (C_2_), propionic/propanoic acid (C_3_), butyric/butanoic acid (C_4_), valeric/pentanoic acid (C_5_), caproic/hexanoic acid (C_6_), enanthic/heptanoic acid (C_7_), caprylic/octanoic acid (C_8_), pelargonic/nonanoic acid (C_9_), capric/decanoic acid (C_10_), undecylic/undecanoic acid (C_11_), and lauric/dodecanoic acid (C_12_). For the acids with a melting point above room temperature (i.e., C_10_, C_11_, and C_12_), the oil-surfactant-acid mixture was heated to 45°C to melt the crystalline, vortexed, and allowed cooldown back to room temperature.

For preparation of W/O emulsions, the oil-surfactant stock solution (without organic acid) was blended with 1% v/v distilled water using a high-shear mixer (M133/1281–0, Biospec Products, Inc., ESGC, Switzerland) at room temperature for 2 min. The obtained coarse W/O emulsions were further homogenized with a microfluidizer (M-110 L, Microfluidics, Newton, MA) at 12 kpsi for 2 passes. The obtained fine W/O emulsions were mixed with organic acids as previously described to make acidified W/O emulsions at 200 mM as calculated based on the sum of the volumes of the water, oil, surfactant, and acid. Analysis of emulsion particle/droplet size was performed with dynamic light scattering (DLS) using the Zetasizer Nano ZS (Malvern Instruments, Worcestershire, United Kingdom), with the results reported as the intensity-weighted mean diameter, Z average. Prior to DLS measurements, the samples were diluted 1:100 with hexadecane (refractive index = 1.434, viscosity = 3.13 mPa·s at 22°C) to prevent multi-scattering (Yi et al., 2014). The 1% water in W/O emulsions was selected based on a previous report determining the required water concentration that achieves antimicrobial enhancement (>6.5 MLR) of the acetic acid-acidified oil (Chuang et al., 2023).

#### 2.3.2. Osmotic pressure assay

Glycerol was used to reduce the water activity (a_w_) of W/O emulsions. For preparation, the oil-surfactant stock solution (without acid) was blended with 1% v/v distilled water and 3% v/v glycerol, homogenized by microfluidization, and mixed with organic acids as previously described. This allowed the antimicrobial efficacy of acidified W/O emulsions to be evaluated at a constant 1% water with reduced a_w_. A dewpoint a_w_ meter (AquaLab Series 3, v2.3, METER Group, Pullman, WA) was used in continuous mode at 22°C for a_w_ measurements. The mean of two consecutive readings within ±0.002 was reported (Decagon Devices, Inc, 2015). The concentration of glycerol was selected based upon different water-glycerol fractions to achieve a certain a_w_ (Nakagawa and Oyama, 2019).

### 2.4. Antimicrobial assay

#### 2.4.1. Treatment specification

A treatment solution (100 μL) was pipetted onto the desiccated cells on coupon, which was transferred to an incubator set at 22°C or 45°C to equilibrate for 5 min and remain holding at the prevailing temperature for 30 min. Upon end of treatment, one coupon was transferred to TSB (10 mL) buffered with 0.25 mM HEPES (pH 7.2, #H4034, Sigma-Aldrich) in a conical polypropylene tube with sterile glass beads (diameter: 1 mm). This was vortexed at 3200 rpm for 2 min to ensure the removal of cells from the coupon to the medium. Serial dilutions were made in 0.1% buffered peptone water (pH 7.2, Difco, Becton Dickinson).

#### 2.4.2. Determination of survival cell numbers

Microbial survival was determined with plate counts on TSAYE with incubation at 37°C for 24 h. Longer incubation times did not result in different viable cell numbers. The detection limit with plate counts was 2 log CFU/coupon. When microbial survival was reduced to below this point, the experiments were repeated with Most Probable Number (MPN).

The MPN determination was performed according to the Bacteriological Analytical Manual (BAM) by the U.S. Food and Drug Administration (Blodgett, 2010). In this context, transferring one coupon to TSB (10 mL) upon end of treatment was considered as a 1:10 dilution. From here, inocula of 1, 0.1 and 0.01 mL were grown in TSB (20 mL/tube) with three tubes each. This allowed MPN determination per coupon to match the MPN/mL digits listed on the BAM table reference where inocula of 0.1, 0.01 and 0.001 mL were used. Presumptive growth was indicated by turbidity after incubating the tubes at 37°C for 24 h. Confirmatory tests were made by plating the turbid cultures on Xylose Lysine Deoxycholate (XLD) agar (#R459902, Fisher Scientific) with incubation at 37°C for 24 h. When all three tubes at each dilution were negative (0, 0, 0), the outcome was interpreted as less than the outcome with all negative tubes at the two lowest dilutions and one positive tube at the highest dilution (0, 0, 1). Thus, the detection limit was 3 MPN/coupon (0.48 log MPN/coupon). Microbial log reduction (MLR) was calculated with base 10:

(1) $$\mathrm{MLR}=\log\;\left(N_{0}/N\right)=\log\;N_{0}-\log\;N$$

where *N_0_* is the viable count of desiccated cells recovered from coupon, and *N* stands for the CFU or MPN survival cell number after treatment. When *N_0_* equals *N*, the treatment was interpreted as not antimicrobial (NA).

### 2.5. Fluorescence lifetime microscopy

#### 2.5.1. Sample preparation

Cell staining was performed in dark following the procedure by Mika et al. with modifications (Mika et al., 2016). For preparation of hydrated cells, an isolated colony was transferred from the working culture to TSB (20 mL) and incubated at 37°C for 18 h. On the day of experiment, this culture was diluted 1:100 with fresh TSB (to 0.02 OD_600_), supplemented with BODIPY FL C_12_ (4,4-difluoro-5,7-dimethyl-4-Bora-3a,4a-diaza-s-indacene-3-dodecanoic acid, #D3822, Fisher Scientific) from a 2.5 mM stock solution in dimethyl sulfoxide (DMSO, #J66650AE, Fisher Scientific) to 2 μM, and incubated at 37°C without shaking for 2–3 h to allow staining. Upon end of incubation, the stained cells were washed twice with dye-free TSB and resuspended in distilled water. A loopful of cells were spread onto a glass bottom dish with poly-d-lysine coating (#P35GC-1.5-10-C, MatTek, Ashland, MA), briefly dried (15 min) in a biosafety cabinet at room temperature, and observed under a microscope. For preparation of desiccated cells, the cells grown on TSAYE plates as lawns were suspended in TSB (to 0.02 OD_600_), mixed with BODIPY, and incubated as previously described. The stained cells were washed and resuspended in distilled water, from which droplets (20 μL) were added to glass bottom dishes and held in a desiccator at 75% ERH at room temperature for 20 h. The concentration of DMSO during staining was 0.08% v/v. The growth of *Salmonella* was not influenced by BODIPY and DMSO at the specified concentrations (Supplementary Figure S1).

#### 2.5.2. FLIM measurement

Fluorescence lifetime imaging microscopy (FLIM) was performed using a confocal microscope (A1/Ti-E inverted, Nikon, Japan) to which an A1-FLIM module (Becker & Hickl, Berlin, Germany) was attached. Glass bottom dishes were loaded with bacterial samples and mounted on a microscope stage top chamber (H301-MINI, Okolab, Sewickley, PA) which was connected to a controlled heating system (Okolab) at the Light Microscopy Facility, Institute for Applied Life Sciences, University of Massachusetts-Amherst. For temperature elevation assays, the chamber was heated to 45°C and the samples were equilibrated for 5 min prior to FLIM measurements. A wavelength of 488 nm (50 mHz) was used to excite BODIPY FL C_12_. Pinhole was 1.0 Airy Unit. Dwell time was 2.2 μs/pixel. Image acquisition was set to a size of 1,024 × 1,024 and measured over 30 s. Emitted photons were detected using a time-correlated single photon counting (TCSPC) module (HPM-100-40 detector, SPC-150 N module card, Becker & Hickl) with a 525/50 bandpass filter (peak of BODIPY FL C_12_ emission spectrum: 511 nm). Each sample was measured for less than 1 h to prevent photobleaching. Single-cell images were obtained by cropping the captured field using the NIS-Elements software (Nikon). Cell-cluster images were shown with the entire captured field.

#### 2.5.3. FLIM data analysis

Analysis of FLIM data was performed using the SPCImage software (v8.6, Becker & Hickl). Regions of interest (ROIs) were drawn around isolated single cells identified in the captured FLIM images. This was followed by binning all the pixels within the respective ROI. The instrument response function (IRF) was automatically defined by the software and approximately 10^4^ photons were assessed per decay curve. This level of photon number is required for analyzing double-exponential decays (Warren et al., 2013). Fitting data with a double-exponential function showed Chi^2^ values ranging from 0.96 to 1.58, indicating a good fit between the observed values and the model predictions. Below is the general expression of a double-exponential decay:

(2) $$f\left(t\right)=a_{1}e^{-t/\tau_{1}}+a_{2}e^{-t/\tau_{2}}$$

where *f* is fluorescence intensity, *t* is time, 𝜏 is the fluorescence lifetime of the exponential components, and 𝛼 is the amplitude of the exponential components (𝛼_1_ + 𝛼_2_ = 1). The BODIPY-based rotors have two conformations within the cytoplastic membrane, which are accompanied by different sensitivities to viscosity due to the molecular orientation (Dent et al., 2015). The long lifetime component derives from the rotor that localizes in the hydrocarbon tail region of the lipid bilayer, and thus is viscosity-sensitive, providing a better representation of the viscosity of ordered membrane structures (Mika et al., 2016). Thus, 𝜏_1_ was used to calculate membrane viscosity.

#### 2.5.4. Calibration of lifetime against viscosity

The measured fluorescence lifetime was used for calculation of membrane viscosity using a lifetime-viscosity calibration funtion:

(3) $$\log\mu =\left(\log\tau +0.75614\right)/0.4569$$

where μ is viscosity in mPa·s (cP) and 𝜏 is fluorescence lifetime in nanosecond. This equation was developed by Hosny et al. by measuring the fluorescence lifetime of BODIPY C_10_ in methanol-glycerol mixtures (bulk liquids of known viscosity) (Hosny et al., 2016). Despite a difference in the hydrocarbon chain-length, the BODIPY-based C_10_ and C_12_ rotors possess identical photophysical properties, such as the viscosity-dependent fluorescence lifetime (Kuimova et al., 2008; Wu et al., 2013; Polita et al., 2020). Thus, Equation (3) was used for lifetime-viscosity calibration of BODIPY FL C_12_ in *Salmonella* membrane.

### 2.6. Statistical analysis

Experiments were conducted in triplicate independently. Two-way analysis of variance (ANOVA) was performed with post-hoc Tukey for pairwise comparison using the Prism software (v9.5.1, GraphPad, San Diego, CA). Differences were determined statistically significant at *p* < 0.05.

## 3. Results and discussion

### 3.1. Formulation screening with *Salmonella* cocktail

**Figure 1:**
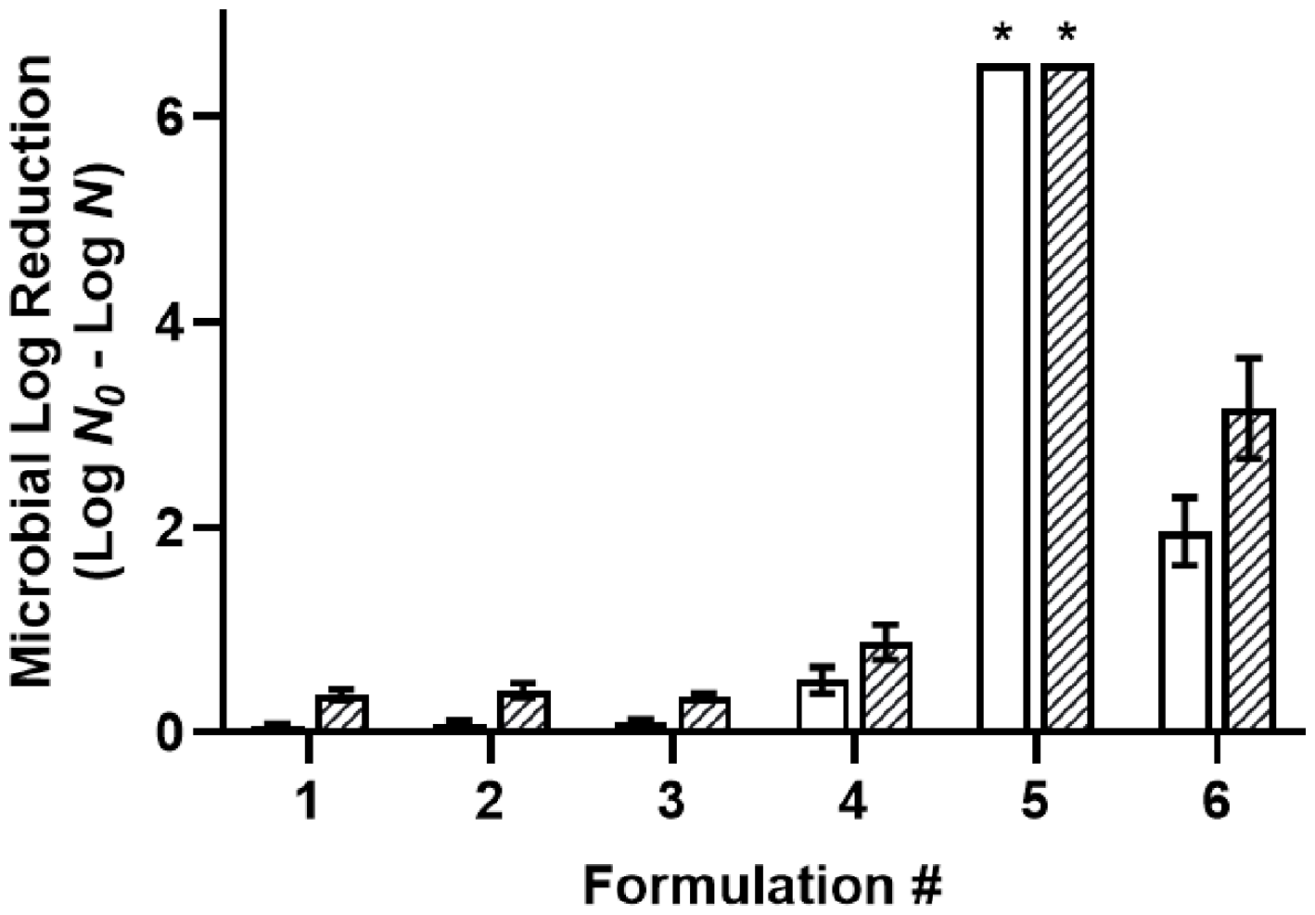
Efficacy of oil-based formulations against a five-strain *Salmonella* cocktail desiccated to 75% ERH, with a 30-min treatment at 22°C (white) or 45°C (striped). Formulations: (1) oil with 3% w/w PGPR surfactant, (2) W/O emulsion with 3% w/w PGPR and 1% v/v distilled water, (3) W/O emulsion with 3% w/w PGPR, 1% v/v distilled, and 3% v/v glycerol, (4) acidified oil with 200 mM acetic acid, and 3% w/w PGPR, (5) acidified W/O emulsion with 200 mM acetic acid, 3% w/w PGPR, and 1% v/v distilled water, (6) acidified W/O emulsion with 200 mM acetic acid, 3% w/w PGPR, 1% v/v distilled water, and 3% v/v glycerol. *Microbial survival was reduced to below the detection limit and expressed as >6.5 MLR/coupon.

The desiccated cells of a four-strain *Salmonella* cocktail were used for screening the antimicrobial efficacy of oil-based formulations (**F1-6**) with a 30-min treatment time (Figure 1). Acetic acid (C_2_) was selected for use in the screening procedure based on our previous report that it was the most effective food-grade acid against desiccated *Salmonella* among different chain-length organic acids (Ghoshal et al., 2022). The use of 1% water in W/O emulsions was based upon another report determining the required water level for acidified W/O emulsions to achieve >6.5 MLR with 200 mM acetic acid (Chuang et al., 2023). Likewise, 3% glycerol was used in **F3** and **F6** to decrease the solution a_w_ while maintaining a constant 1% water. The results showed that the non-acidified controls, including oil with surfactant (**F1**), oil with a W/O emulsion (**F2**), and oil with a W/O emulsion with glycerol (**F3**) had little to no antimicrobial efficacy. Dissolving 200 mM acetic acid (C_2_, **F4**) in oil without water showed low levels of antimicrobial efficacy with 0.51 and 0.88 MLR at 22°C and 45°C, respectively. Dispersing 1% water within the acidified oil as a W/O emulsion (**F5**) increased the solution a_w_ from 0.33 to 0.92 and the antimicrobial efficacy greatly increased compared to without water (**F4**) with a >6.5 MLR at both 22°C and 45°C. Adding glycerol to the acidified oil with a W/O emulsion (**F6**) reduced the solution a_w_ from 0.92 to 0.38 and the antimicrobial efficacy decreased compared to the emulsion without glycerol (**F5**). The acidified oil with a W/O emulsion with glycerol (**F6**) showed 1.96 and 3.16 MLR at 22°C and 45°C, respectively. The antimicrobial enhancement from water dispersion may be attributed to the partitioning of acetic acid from the oil phase into the aqueous phase which subsequently functioned as a vehicle for the acid to enter the cytoplasm, as depicted in a proposed partition equilibria of organic acids at the oil–water-cell interface (Supplementary Figure S2). Since the antimicrobial efficacy was greatly reduced with the addition of glycerol, these results indicated that the antimicrobial mechanism may be due to differential osmotic pressure. As all the *Salmonella* strains were susceptible to **F5** showing >6.5 MLR at both the tested temperatures, the strain most relevant to LMF processing (*S.* Enteritidis) was used as the representative strain in all subsequent experiments.

### 3.2. Influences of organic acid carbon chain-length, treatment temperature, and solution a_w_ on the efficacy of acidified W/O emulsions against desiccated *Salmonella*

Formulations comprising different chain-length acids were tested against desiccated *S.* Enteritidis with a 30-min treatment time (Table 1). The measured solution a_w_ of acidified oil was 0.51 with C_1_ and 0.33 with the remaining acids (C_2-12_), that of acidified W/O emulsion was 0.92 for all the acids, and that of acidified W/O emulsion with glycerol was 0.38 for all the acids (Supplementary Table S1). Acidified oils formulated with the shorter chain-length acids (C_1-3_) were found more effective than those formulated with the longer chain-length acids (C_4-12_). The antimicrobial efficacy of C_1_ acidified oil was pronounced, showing >6.5 MLR at both 22°C and 45°C. The C_2_ acidified oil showed 0.69 MLR at 22°C and 1.42 MLR at 45°C. The C_3_ acidified oil showed 0.89 MLR at 22°C and 1.11 MLR at 45°C. By comparison, the C_4-12_ acidified oils showed little to no MLR at 22°C which slightly increased at 45°C. These increments with temperature elevation were likely due to the sole influence of heating as previously illustrated with the non-acidified controls (F1-3, Figure 1).

**Table 1:** Influence of organic acid carbon chain-length and treatment temperature on the efficacy of acidified oils and W/O emulsions against desiccated *Salmonella* Enteritidis.

Organic acid*^a^*	Chain length	Delivery system and treatment temperature (MLR ± SD)*^b^*					
		Oil with PGPR		W/O emulsion		W/O emulsion with glycerol	
		22°C	45°C	22°C	45°C	22°C	45°C
Formic	C_1_	>6.52*^B^*	>6.52*^B^*	>6.52*^B^*	>6.52*^B^*	1.32 ± 0.65*^A^*	>6.52*^B^*
Acetic	C_2_	0.69 ± 0.19*^A^*	1.42 ± 0.14*^B^*	>6.52*^D^*	>6.52*^D^*	0.81 ± 0.24*^A^*	2.53 ± 0.75*^C^*
Propionic	C_3_	0.89 ± 0.24*^A^*	1.11 ± 0.27*^AB^*	>6.52*^C^*	>6.52*^C^*	0.73 ± 0.22*^A^*	1.41 ± 0.66*^B^*
Butyric	C_4_	NA	0.52 ± 0.13*^A^*	0.55 ± 0.06*^A^*	>6.52*^C^*	0.31 ± 0.16*^A^*	1.40 ± 0.31*^B^*
Valeric	C_5_	NA	0.31 ± 0.02*^A^*	0.11 ± 0.05*^A^*	>6.52*^C^*	NA	1.12 ± 0.16*^B^*
Caproic	C_6_	0.21 ± 0.09*^A^*	0.68 ± 0.08*^B^*	0.72 ± 0.18*^B^*	>6.52*^C^*	0.10 ± 0.03*^A^*	0.74 ± 0.48*^B^*
Enanthic	C_7_	NA	0.34 ± 0.03*^A^*	0.34 ± 0.26*^A^*	>6.52*^C^*	NA	0.79 ± 0.22*^A^*
Caprylic	C_8_	0.26 ± 0.18*^A^*	0.49 ± 0.26*^AB^*	0.94 ± 0.26*^B^*	>6.52*^C^*	0.56 ± 0.18*^AB^*	0.74 ± 0.11*^B^*
Pelargonic	C_9_	NA	0.39 ± 0.11*^A^*	0.31 ± 0.10*^A^*	>6.52*^B^*	NA	0.30 ± 0.09*^A^*
Capric	C_10_	0.23 ± 0.04*^A^*	0.28 ± 0.09*^A^*	0.67 ± 0.17*^A^*	>6.52*^B^*	0.37 ± 0.06*^A^*	0.57 ± 0.09*^A^*
Undecylic	C_11_	NA	0.31 ± 0.03*^A^*	0.38 ± 0.09*^A^*	>6.52*^B^*	NA	0.22 ± 0.18*^A^*
Lauric	C_12_	0.16 ± 0.06*^A^*	0.40 ± 0.17*^A^*	1.05 ± 0.28*^B^*	>6.52*^C^*	0.49 ± 0.12*^A^*	1.02 ± 0.04*^B^*

Different uppercase letters indicated significant differences (*p* < 0.05) within each row. Comparison was not drawn where treatment was not antimicrobial (NA).

a The organic acid concentration was 200 mM based upon the final solution volume.

b A 30-min contact time against cells desiccated to 75% ERH.

Dispersing 1% water within acidified oils as an emulsion enhanced the antimicrobial efficacy depending on the acid carbon chain-length and treatment temperature. Acidified W/O emulsions formulated with C_1-3_ acids showed >6.5 MLR at both 22°C and 45°C. However, those formulated with C_4-12_ acids showed little to no MLR at 22°C but were enhanced at 45°C (>6.5 MLR). All the emulsions were stable in terms of droplet size after a 30-min incubation at 45°C (Supplementary Table S2), and thus we do not believe the antimicrobial enhancement by temperature elevation was related to emulsion instability. Glycerol attenuated the antimicrobial efficacy of C_2_ and C_3_ acidified W/O emulsions at both 22°C and 45°C, and attenuated that of C_4-12_ acidified W/O emulsions at 45°C, to MLR levels close to the corresponding acidified oils. Such efficacy attenuation aligned with the reduced solution a_w_ upon glycerol addition. It appeared C_1_ was an exception, that the a_w_ of acidified oil was greater than that of the acidified W/O emulsion with glycerol (Supplementary Table S1), which was in line with the corresponding MLR data (Table 1). However, the difference between the antimicrobial efficacy of C_1_ and C_2_ acidified oils was pronounced, indicating that the solution a_w_ measured at 22°C only partially explained the mechanism. Other factors may have been involved, e.g., the a_w_ of oil is a function of the prevailing temperature (Yang et al., 2020; Xie et al., 2021). Thus, the a_w_ of acidified oils formulated with different organic acids could exhibit different temperature dependence, thereby showing varying levels of antimicrobial efficacy. In addition, the data with C_4-12_ acidified W/O emulsions suggested there may be a correlation between treatment temperature and the acid carbon chain-length. We hypothesize that heating may result in changes in the ability of the longer chain-length acids to permeate the cells, possibly due to changes in the viscosity of the cellular membrane.

### 3.3. Membrane viscosity of *Salmonella* upon heating

**Figure 2:**
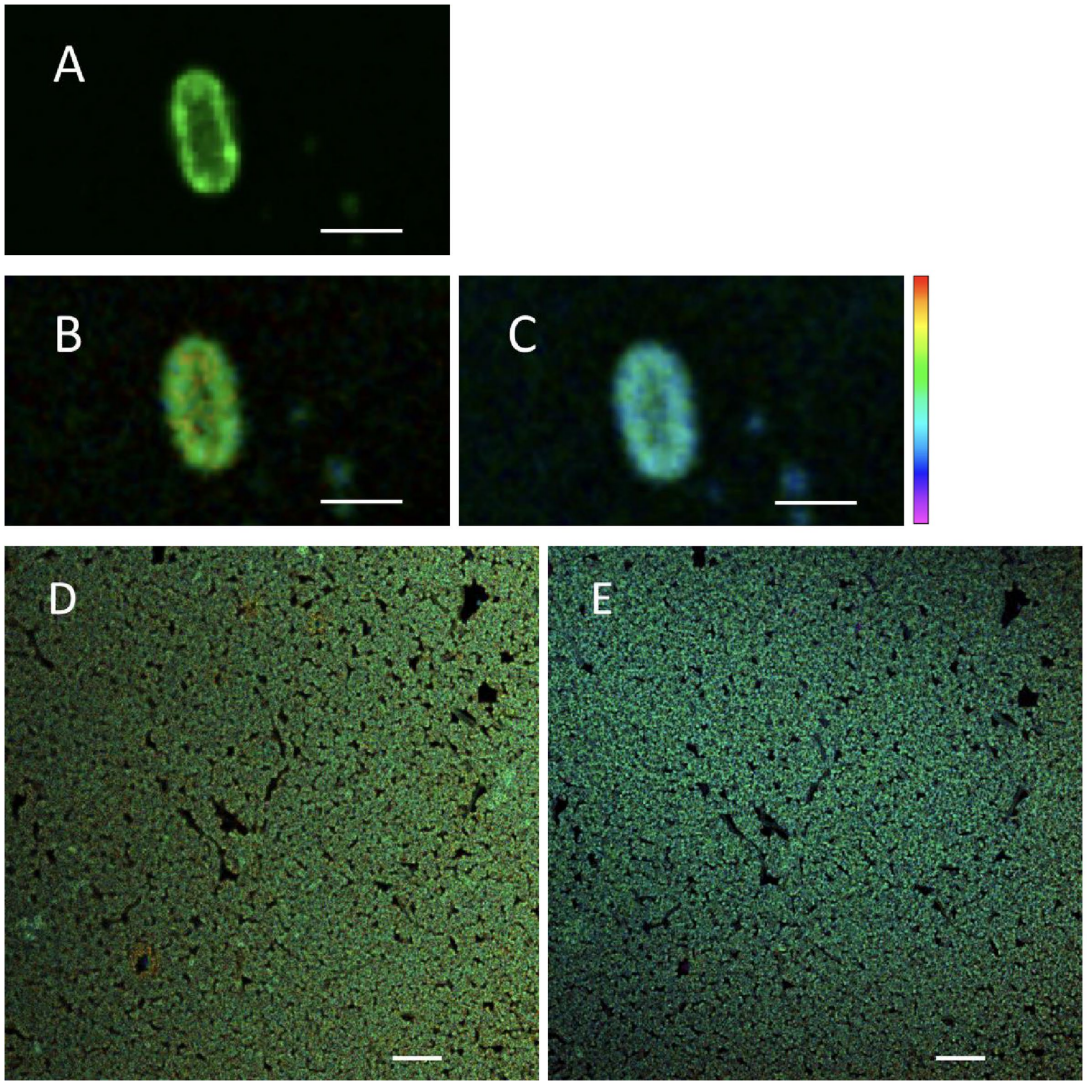
Fluorescent micrographs of hydrated *Salmonella* Enteritidis stained with BODIPY FL C_12_: confocal scanned **(A)**, FLIM measured at 22°C **(B,D)**, and FLIM measured at 45°C **(C,E)**. Scale bar: 2 μm [**(A–C)**, single cell]; 10 μm [**(D,E)**, cell cluster]. Of the lifetime color scale, the upper (red) and lower (purple) limits were 5.5 and 3 ns, respectively.

To test the hypothesis that changing treatment temperature can change the cellular membrane viscosity, FLIM was used to assess the fluorescence lifetime of BODIPY-stained *S.* Enteritidis (Figure 2). The confocal micrograph showed a ring-like fluorescent pattern at the periphery of the stained cells (Figure 2A), indicating rotor localization in the plasma membrane, in agreement with the observation by Nenninger et al. that BODIPY-based rotors stained the plasma membrane of vegetative bacterial cells producing a fluorescent halo (Nenninger et al., 2014). The FLIM images were shown with a single cell (Figures 2B,C) and as a cluster (Figures 2D,E). A decrease in fluorescence lifetime was observed with increasing temperature. The measured fluorescence lifetime values of hydrated cells were 4.46 ns at 22°C and 4.24 ns at 45°C, which were calculated to viscosity values of 1,199 mPa·s at 22°C and 1,082 mPa·s at 45°C. These values are comparable with the existing literature. Oswald et al. measured the protein diffusion coefficients through the membrane of hydrated *Escherichia coli* cells and fitted the values with the Saffman-Delbrück model, reporting a membrane viscosity value of 1,200 mPa·s at 23°C (Oswald et al., 2016). Mika et al. reported that, with the FLIM-BODIPY approach, the membrane viscosity of hydrated *E. coli* cells was 1,160 mPa·s at 23°C which decreased to 950 mPa·s at 37°C (Blodgett, 2010).

**Figure 3:**
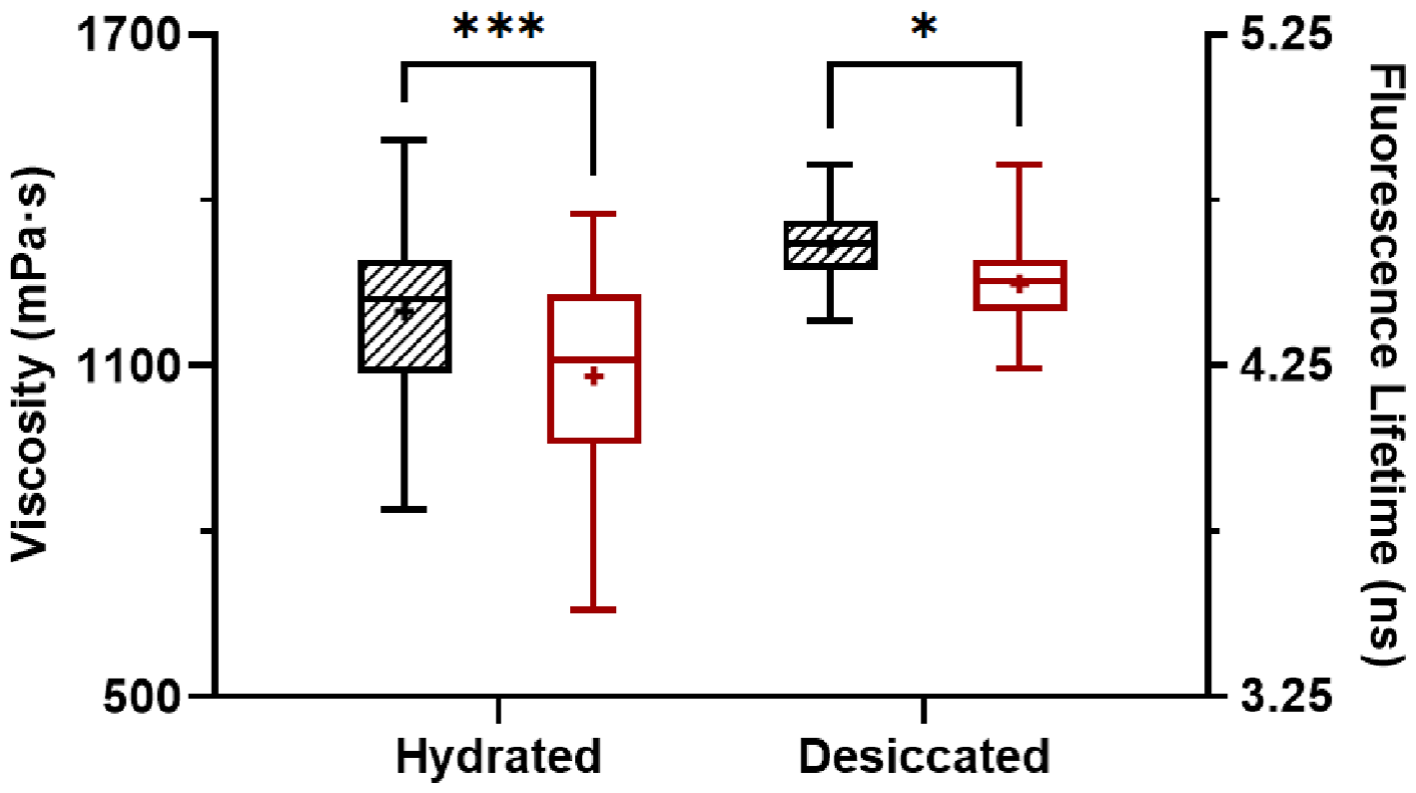
Membrane viscosity of hydrated and desiccated *Salmonella* Enteritidis at 22°C (striped) and 45°C (white). The FLIM data was obtained from 37 hydrated cells and 49 desiccated cells. The same cells were used for measurements at 22°C and 45°C. Within each data set, whiskers (error bars) indicated the maximum and minimum values, the box covered the interquartile range (25–75th percentile), the horizontal line in the box was the median, and the plus mark was the mean. Differences were significant at *p* < 0.05 (*) and < 0.001 (***).

After desiccation to 75% ERH, *S.* Enteritidis cells had increased membrane viscosity (Figure 3). At room temperature (22°C), the mean of the calculated membrane viscosity values of hydrated cells was 1,199 mPa·s, while after desiccation to 75% ERH, the mean membrane viscosity increased to 1,309 mPa·s. This was also observed at 45°C, with a mean membrane viscosity of 1,082 mPa·s for hydrated cells and after desiccation to 75% ERH, the mean membrane viscosity increased to 1,245 mPa·s. Temperature elevation to 45°C significantly decreased the membrane viscosity for both cellular hydration levels, and the difference was found more pronounced for the hydrated cells (*p* < 0.001) than the desiccated cells (*p* < 0.05). *Salmonella* exhibits cross-tolerance to multiple environmental stresses following exposure to one stress (Gruzdev et al., 2011; Ye et al., 2019; Datta et al., 2020). In particular, the thermal resistance of desiccated *Salmonella* is pronounced. One proposed mechanism is that the fluidity of cellular membrane can vary upon environmental conditions, thereby affecting thermodynamics (Álvarez-Ordóñez et al., 2008, 2009; Stein, 2012; Ernst et al., 2016; Chen and Jiang, 2017).

Based upon our results, we propose that the increased antimicrobial efficacy of C_4-12_ acidified W/O emulsions at 45°C (Table 1), may be due to a lower membrane viscosity upon temperature elevation (Figure 3). This decrease in membrane viscosity could allow longer chain-length acids (C_4-12_) to diffuse into the cellular membrane causing damage. The damaged membrane in combination with the water in the W/O emulsion contributes to cellular hypoosmotic stress resulting in cellular death at the elevated temperature. The addition of glycerol to the emulsions (Table 1) reduces the antimicrobial efficacy, confirming the involvement of water stress in cellular destruction.

In conclusion, we have found that the antimicrobial efficacy of organic acids within W/O emulsion systems is dependent upon acid chain-length and treatment temperature, with the longer chain acids (C_4-12_) becoming greatly antimicrobial when combined with elevated temperature. We have shown that the cellular membrane becomes less viscous at elevated temperatures and likely contributes to the increase in antimicrobial efficacy. However, it is of importance to note a caveat that the temperature-viscosity relationship does not fully explain why some fatty acids that are much smaller than the lipids in bacterial membrane, such as butyric acid, did not show antimicrobial efficacy without temperature elevation.

## Data availability statement

The original contributions presented in the study are included in the article/Supplementary material, further inquiries can be directed to the corresponding author.

## Author contributions

SC: methodology, acquisition, analysis interpretation of data, drafting, and editing. MG: methodology development. LM: conception of project, data analysis drafting, editing and revising, and approval for publication. All authors contributed to the article and approved the submitted version.

## Funding

This work is based upon work supported by the National Institute of Food and Agriculture (NIFA), U.S. Department of Agriculture (USDA), and the Center for Agriculture, Food, and the Environment and the Department of Food Science at the University of Massachusetts—Amherst, under project number MAS00567, with support from the Foundational and Applied Science Program (grant number 2020-67017-30786) of the USDA—NIFA.

## Conflict of interest

The authors declare that the research was conducted in the absence of any commercial or financial relationships that could be construed as a potential conflict of interest.

## Publisher’s note

All claims expressed in this article are solely those of the authors and do not necessarily represent those of their affiliated organizations, or those of the publisher, the editors and the reviewers. Any product that may be evaluated in this article, or claim that may be made by its manufacturer, is not guaranteed or endorsed by the publisher.
