# Supplementary material for: Efficacy of acidified water-in-oil emulsions against desiccated Salmonella as a function of acid carbon chain-length and membrane viscosity
Source: Front Microbiol. 2023 Jun 12;14:1197473. doi: 10.3389/fmicb.2023.1197473 (PMC10291884; doi:10.3389/fmicb.2023.1197473)
Supplement: Supplementary file 1 [file Data_Sheet_1.docx]

Supplementary Material

**Efficacy of Acidified Water-in-Oil Emulsions against Desiccated *Salmonella* as A Function of Acid Carbon Chain-Length and Membrane Viscosity**

**Shihyu Chuang, Mrinalini Ghoshal, Lynne McLandsborough***

*** Correspondence:** Lynne McLandsborough: [lm@foodsci.umass.edu](mailto:lm@foodsci.umass.edu)

**Supplementary Table 1.** Measured water activity (a_w_) of acidified oils, W/O emulsions, and W/O emulsions with glycerol, formulated with different carbon chain-length organic acids.

| **Organic  Acid***^a^* | **Chain  Length** | **Measured Water Activity***^b^* | | |
| --- | --- | --- | --- | --- |
|  |  | **Oil with PGPR** | **W/O Emulsion** | **W/O Emulsion  with Glycerol** |
| Formic | C_1_ | 0.51 | 0.92 | 0.38 |
| Acetic | C_2_ | 0.33 | 0.92 | 0.38 |
| Propionic | C_3_ | 0.33 | 0.92 | 0.38 |
| Butyric | C_4_ | 0.33 | 0.92 | 0.38 |
| Valeric | C_5_ | 0.33 | 0.92 | 0.38 |
| Caproic | C_6_ | 0.33 | 0.92 | 0.38 |
| Enanthic | C_7_ | 0.33 | 0.92 | 0.38 |
| Caprylic | C_8_ | 0.33 | 0.92 | 0.38 |
| Pelargonic | C_9_ | 0.33 | 0.92 | 0.38 |
| Capric | C_10_ | 0.33 | 0.92 | 0.38 |
| Undecylic | C_11_ | 0.33 | 0.92 | 0.38 |
| Lauric | C_12_ | 0.33 | 0.92 | 0.38 |

*^a^* The organic acid concentration was 200 mM based upon the final solution volume.

*^b^* The solution a_w_ was measured with dewpoint method at 22 °C**.**

**Supplementary Table 2.** Dynamic light scattering (DLS) analysis of acidified W/O emulsions subjected to heating at 45 °C for 30 min.

| **W/O Emulsions***^a^* **with Different Carbon Chain-Length (C_n_) Organic Acids** | **Mean Particle Diameter (Z average, μm)** | |
| --- | --- | --- |
|  | **Upon Formation (22 °C)** | **After Heating (45 °C)***^b^* |
| **C_1_** | 0.6 ± 0.1 | 1.0 ± 0.3 |
| **C_2_** | 0.7 ± 0.2 | 1.0 ± 0.3 |
| **C_3_** | 0.8 ± 0.1 | 0.8 ± 0.2 |
| **C_4_** | 0.7 ± 0.2 | 0.7 ± 0.1 |
| **C_5_** | 0.7 ± 0.1 | 0.7 ± 0.3 |
| **C_6_** | 0.8 ± 0.3 | 1.0 ± 0.3 |
| **C_7_** | 0.6 ± 0.1 | 0.8 ± 0.3 |
| **C_8_** | 0.9 ± 0.4 | 0.9 ± 0.3 |
| **C_9_** | 0.6 ± 0.1 | 0.9 ± 0.3 |
| **C_10_** | 1.0 ± 0.3 | 0.9 ± 0.3 |
| **C_11_** | 0.7 ± 0.3 | 0.6 ± 0.2 |
| **C_12_** | 0.9 ± 0.4 | 0.9 ± 0.2 |

Differences in droplet size were not significant (*P* > 0.05) among all the emulsions, upon formation and after heating.

*^a^* The W/O emulsions were prepared via microfluidization at 12 kpsi for 2 passes (3% w/w PGPR, 1% v/v distilled water), followed by blending with C_1-12_ acids to 200 mM.

*^b^* The samples were equilibrated at 45 °C for 5 min and remained holding for 30 min.


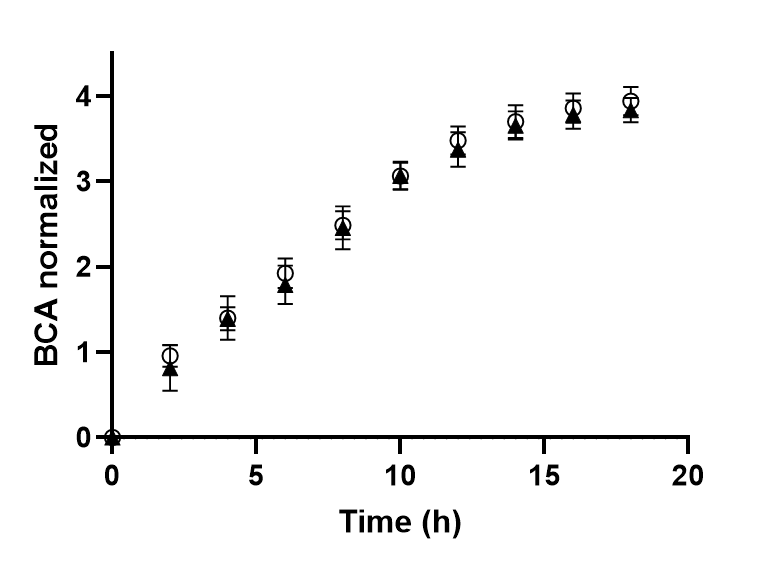


**Supplementary Figure 1.** Growth of *Salmonella* Enteritidis at room temperature in TSB (open circle) in the presence of 2 μM BODIPY FL C_12_ and 0.08% v/v DMSO (closed triangle). An overnight culture was diluted with fresh medium to approximately 10^6 CFU/mL and added with BODIPY from a DMSO stock to the specified concentrations. Aliquots were added to multiwell plates for subsequent measurements with the oCelloScope (BioSense Solutions, Farum, Denmark) following the procedure by Ghoshal et al. (2022). Microbial growth was analyzed using the Background Corrected Absorption (BCA) Normalized algorithm with a dedicated software (UniExplorer, v10.1, BioSense Solutions).

**
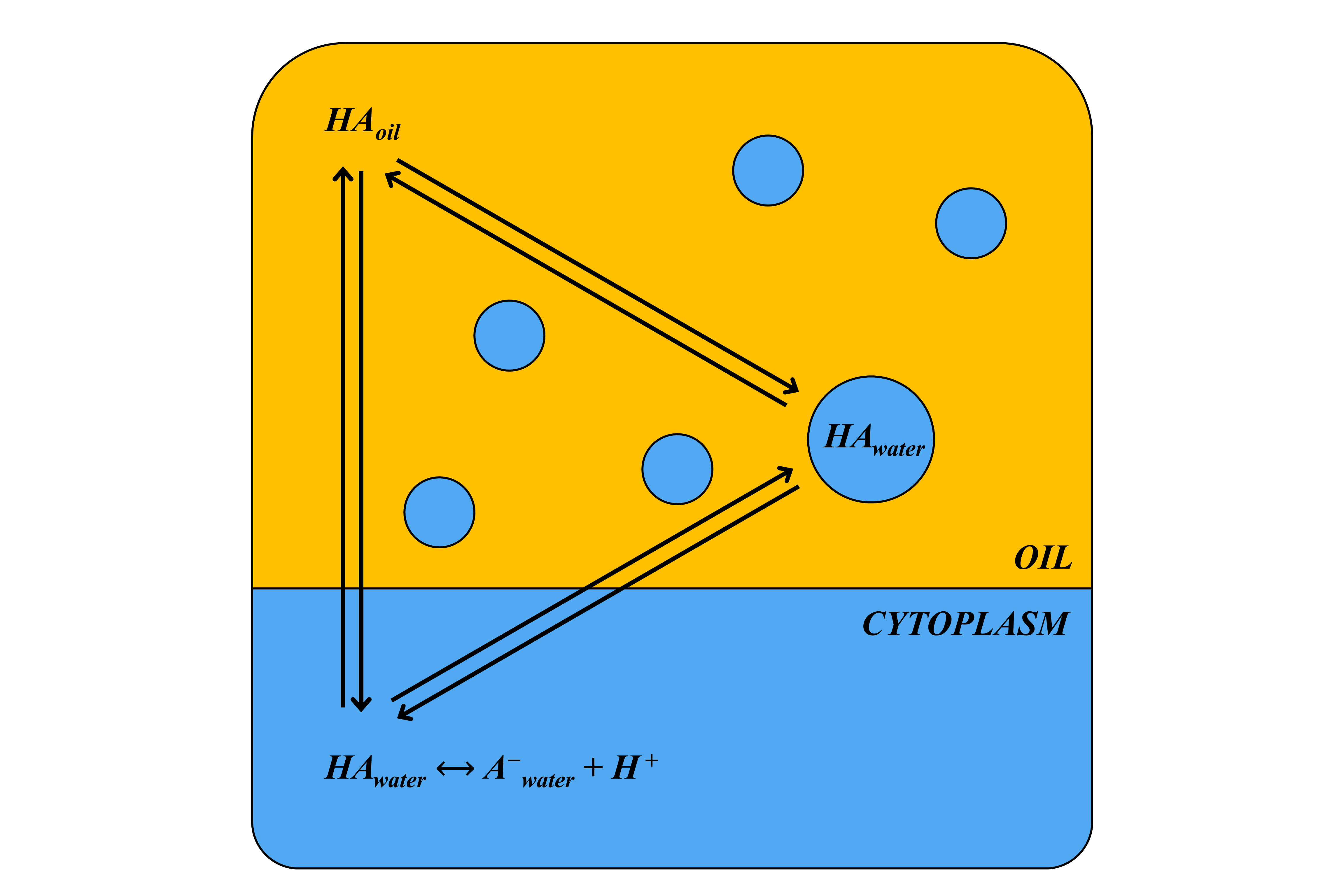
**

**Supplementary Figure 2.** Oil-water-cell partition equilibria of organic acids. Depicted are the partitioning of undissociated organic acids among the three phases, and the ionization of organic acids within the neutral cytoplasm.
